# Distributional records of Ross Sea (Antarctica) planktic Copepoda from bibliographic data and samples curated at the Italian National Antarctic Museum (MNA): checklist of species collected in the Ross Sea sector from 1987 to 1995

**DOI:** 10.3897/zookeys.969.52334

**Published:** 2020-09-17

**Authors:** Guido Bonello, Marco Grillo, Matteo Cecchetto, Marina Giallain, Antonia Granata, Letterio Guglielmo, Luigi Pane, Stefano Schiaparelli

**Affiliations:** 1 Italian National Antarctic Museum (MNA, Section of Genoa), University of Genoa, Genoa, Italy; 2 Department of Earth, Environmental and Life Science (DISTAV), University of Genoa, Genoa, Italy; 3 Department of Chemical, Biological, Pharmaceutical and Environmental Sciences (ChiBioFarAm), University of Messina, Messina, Italy; 4 Stazione Zoologica Anton Dohrn (SZN), Villa Pace, Messina, Italy

**Keywords:** abundance, biogeography, BIONESS, distribution, museum collection, Terra Nova Bay

## Abstract

Distributional data on planktic copepods (Crustacea, Copepoda) collected in the framework of the III^rd^, V^th^, and X^th^ Expeditions of the Italian National Antarctic Program (PNRA) to the Ross Sea sector from 1987 to 1995 are here provided. Sampling was performed with BIONESS and WP2 nets at 94 sampling stations at depths of 0–1,000 m, with a special focus on the Terra Nova Bay area. Altogether, this dataset comprises 6,027 distributional records, out of which 5,306 were obtained by digitizing original data reports and 721 are based on physical museum vouchers curated by the Italian National Antarctic Museum (MNA, Section of Genoa). The MNA samples include 8,224 individual specimens that were identified to the lowest possible taxonomic level. They belong to four orders, 25 families, 52 genera, and 82 morphological units (out of which 17 could be determined at the genus level only). A variety of environmental data were also recorded at each of the sampling stations, and we report original abundances (ind/m^3^) to enable future species distribution modelling. From a biogeographic point of view, the distributional data here reported represented new records for the Global Biogeographic Information Facility (GBIF) registry. In particular, 62% of the total number of species are new records for the Ross Sea sector and another 28% new records for the Antarctic region.

## Introduction

The study of planktic copepods in the Ross Sea represented one of the earliest scientific efforts and targets of the first oceanographic expeditions of the Italian National Antarctic Research Program (PNRA), which started in 1985. One of the underlying reasons for this dedication was the fact that, at the time of sampling, there was a general lack of exhaustive and accessible literature about Copepoda for the Ross Sea region. Therefore, specific sampling activities were planned to define the copepod community structure and establish a reference baseline for comparisons with future research findings (Amato 1990).

Copepods are one of the key groups in marine trophic chains, representing up to 70% of the mesozooplanktic biomass, a condition typically found in all Antarctic seas (Carli et al. 2002). Besides their dominance, Antarctic planktic copepods are also important because of their degree of adaptation to the exacerbated seasonality of food availability, which is determined by the polar light regime and sea ice dynamics, both affecting the primary production(Hagen and Schnack-Schiel 1996). This led to a variety of specific physiological and developmental strategies. For example, some pelagic herbivorous copepods synchronize their gonadal and life-stage with phytoplankton blooms to gain the best from the extremely short Antarctic summer months (Hagen and Schnack-Schiel 1996; Minutoli et al. 2017). Other species are intimately linked to the sea ice, which provides an important habitat for small grazers in general but especially copepods (Loots et al. 2009; Pusceddu et al. 2009). Many Antarctic copepods represent a numerically dominant fraction of sea-ice communities and may have annual life cycles which take place completely in, partially within, or underneath the sea ice (Schnack-Schiel et al. 1995, 2001; Tanimura et al. 1996; Swadling 2001, Guglielmo et al. 2007). Other species, such as *Calanoides acutus* (Giesbrecht, 1902), *Calanus propinquus* (Brady, 1883), and *Metridia gerlachei* (Giesbrecht, 1902), are known to accumulate wax esters and triacylglycerols to overcome winter conditions(Schnack-Schiel and Hagen 1994; Hagen et al. 1993; Reinhardt and Van Vleet 1986). Other meso- to bathypelagic carnivorous species such as *Paraeuchaeta antarctica* (Giesbrecht, 1902) (Zmijewska 1993; Mazzocchi et al. 1995) exert an essential ecological and trophic role, being at the same time predators of smaller species belonging to the genera *Oithona*, *Oncaea*, and *Metridia* (Oresland 1991, 1995; Oresland and Ward 1993) and prey for larger macrozooplanktic organisms such as amphipods and mysids (Hopkins 1985), chaetognaths (Oresland 1995), midwater fishes (Williams 1985; Kellermann 1987), and even for benthic organisms such as brittle stars (Dearborn et al. 2011).

During the first Italian oceanographic expeditions in the Ross Sea, it was therefore natural to focus on copepods, and specifically on their distribution (Carli et al. 2000, 2002; Zunini Sertorio et al. 2000) and diversity (Carli et al. 1990, 1992; Zunini Sertorio et al. 1990, 1992).

The present copepod dataset from the Ross Sea is the eighth MNA contribution to the Antarctic Biodiversity Portal, the thematic Antarctic node for both the Ocean Biogeographic Information System (AntOBIS) and the Global Biodiversity Information Facility (ANTABIF) (http://www.biodiversity.aq). Previous MNA contributions focused on Mollusca, Tanaidacea, Fungi, Ophiuroidea, Porifera, Bryozoa, and Rotifera (Ghiglione et al. 2013, 2018; Piazza et al. 2014; Selbmann et al. 2015; Cecchetto et al. 2017, 2019; Garlasché et al. 2020).

This dataset also represents an Italian contribution to the CCAMLR CONSERVATION MEASURE 91-05 (2016) for the Ross Sea region Marine Protected Area, specifically, addressing Annex 91-05/C (“long-term monitoring of benthic ecosystem functions”).

## Project description

**Project title**: Distributional records of Ross Sea (Antarctica) planktic Copepoda from bibliographic data and samples curated at the Italian National Antarctic Museum (MNA): checklist of species collected in the Ross Sea sector from 1987 to 1995.

**Curator and promoter**: Stefano Schiaparelli.

**Personnel**: Bonello Guido, Marco Grillo, Matteo Cecchetto, Marina Giallain, Antonia Granata, Letterio Guglielmo, Luigi Pane, Stefano Schiaparelli.

**Funding**: Data originated in the framework of the first three Italian Antarctic Oceanographic expeditions carried out from 1988 to 1995 within 3 different research projects funded by the PNRA:

– III^rd^ Italian Antarctic expedition (1987/1988), Project: “*Zooplancton – distribuzione spaziale e verticale delle comunità zooplanctoniche nella Baia di Terra Nova* (*Mare di Ross*) *con particolare riferimento al Krill*”; Project code 2.1.4.2.; R/V “*Polar Queen*”; Scientific coordinator: Prof. Letterio Guglielmo.

– V^th^ Italian Antarctic expedition (1989/1990), Project: “*Campagna oceanografica nel mare di Ross*”; R/V “*Cariboo*”; Scientific coordinator: Prof. Letterio Guglielmo.

– X^th^ Italian Antarctic expedition (1994–1995), Project: “*Ecologia zooplancton e micronecton*”; Project code (6.9); R/V ”*Malippo*”; Scientific coordinator: Dr. Riccardo Cattaneo-Vietti

The Italian National Antarctic Museum (MNA) hired two experts, G. Bonello and M. Grillo, with the research contracts #2993 and #2992, respectively, issued on June 25^th^ 2019, to revise plankton collections dating back to the first Italian Expeditions.

### Design description

As the dataset here presented was assembled from data and vouchers collected in the framework of different oceanographic expeditions, which had multiple scientific targets and deployed a variety of sampling gears to investigate the water column physical features and plankton diversity, we briefly introduce the general motivations and scopes of each one of these PNRA expeditions.

The oldest records of the dataset correspond to samples collected during the III^rd^ Italian Antarctic expedition in 1987–1988, only two years after the opening of the Italian research station “Mario Zucchelli” (called “Terra Nova” at that time). This was also the first Italian Antarctic oceanographic expedition, and since there was practically no previous information on the study site (Terra Nova Bay, Ross Sea), the objective of this expedition was to define the spatial and temporal variability of physical, chemical and biological characteristics in this area (Faranda et al. 2000). The line of research on zooplankton developed for this peculiar expedition engaged multiple researchers and pursued the development of a first characterization of the community structure, on the taxonomic, spatial and ecological aspects for the region (Carli et al. 1990; Guglielmo et al. 1990; McKenzie et al. 1990; Zunini Sertorio et al. 1990).

The second oceanographic expedition (V^th^ Italian Antarctic Expedition) took place two years later and investigated a larger geographic area in the Pacific sector of the Southern Ocean. The larger scale of the geographic study site reflected the more ambitious (compared to the first expedition) objectives of the expedition, aiming at achieving a better understanding of the functioning of the Antarctic pelagic ecosystems, through the study of hydrodynamic features (Faranda et al. 2000) in an area characterized by water mass distribution associated with frontal systems. Within this framework, the study of zooplanktic communities would have allowed a better characterization of the effects of these abiotic features on the trophic structure (Benassi et al. 1992; Carli et al. 1992; Guglielmo et al. 1992; Zunini Sertorio et al. 1992).

Finally, the third oceanographic expedition (X^th^ Italian Antarctic Expedition) was carried out during the austral summer of 1994–1995. Most of the sampling was conducted along the 175° meridian, from the northern continental slope to the Ross Sea Ice Shelf. The main purpose of the expedition was to further investigate the effects of the ice-edge retreat on primary production (Faranda et al. 2000). As part of the project, the coastal zooplankton community structure, as well as an evaluation of the biomass and lipid content of the total zooplankton, was examined (Carli et al. 2002; Pane et al. 2004).

This dataset is not only important for the history of Italian research in Antarctica, but, as it dates back to 1987, it also provides a source of historical data, hence representing a useful baseline to measure possible changes and shifts in copepod abundance and diversity in the Ross Sea area that may have occurred in the meantime. At the same time, as the highly ambitious scopes of those expeditions also lead to the production, for the same sampling stations, of an extensive amount of biological and chemical-physical information, as well as about other taxa (Table 1), this copepod dataset can also be used to model and understand species distributions occurring in an environmental setting of more than 30 years ago to be compared with present-day situation.

**Table 1.** Type of sampling and main bibliographic references about the the III^rd^, V^th^, and X^th^ Expeditions of the Italian National Antarctic Program (PNRA).

Type of data	References
**Water column communities**	
Bacterioplankton and heterotrophic bacteria	Bruni et al. 1990
Picoplankton	La Ferla et al. 1992
Phytoplankton	Goffart et al. 1992; Innamorati et al. 1992; Nuccio et al. 1992
Zooplankton	Guglielmo et al. 1990, 1992; Hecq and Guglielmo 1992
Microzooplankton	(Fonda Umani and Monti 1990; Maugeri 1992)
**Physical variables**	
Temperature	Innamorati et al. 1990; Magazzù and Decembrini 1990; Fabiano et al. 1991a
Practical salinity, density excess, and potential temperature	Boldrin and Stocchino 1990
Salinity	Innamorati et al. 1990
Nutrients, dissolved oxygen, pH, total alkalinity, and total inorganic carbon	Catalano and Benedetti 1990; Catalano et al. 1991a, 1991b; Catalano 1992
**Biological variables**	
Particulate organic matter	Fabiano et al. 1991b, 1991c, 1992
Total suspended matter, particulate carbohydrates, proteins, and lipids	Fabiano et al. 1992
Total and fractioned photosynthetic pigments concentration and primary production	Innamorati et al. 1990, 1991; Magazzù and Decembrini 1991, 1992; Saggiomo et al. 1992

## Methods

### Study extent and sampling description

**Figure 1.**
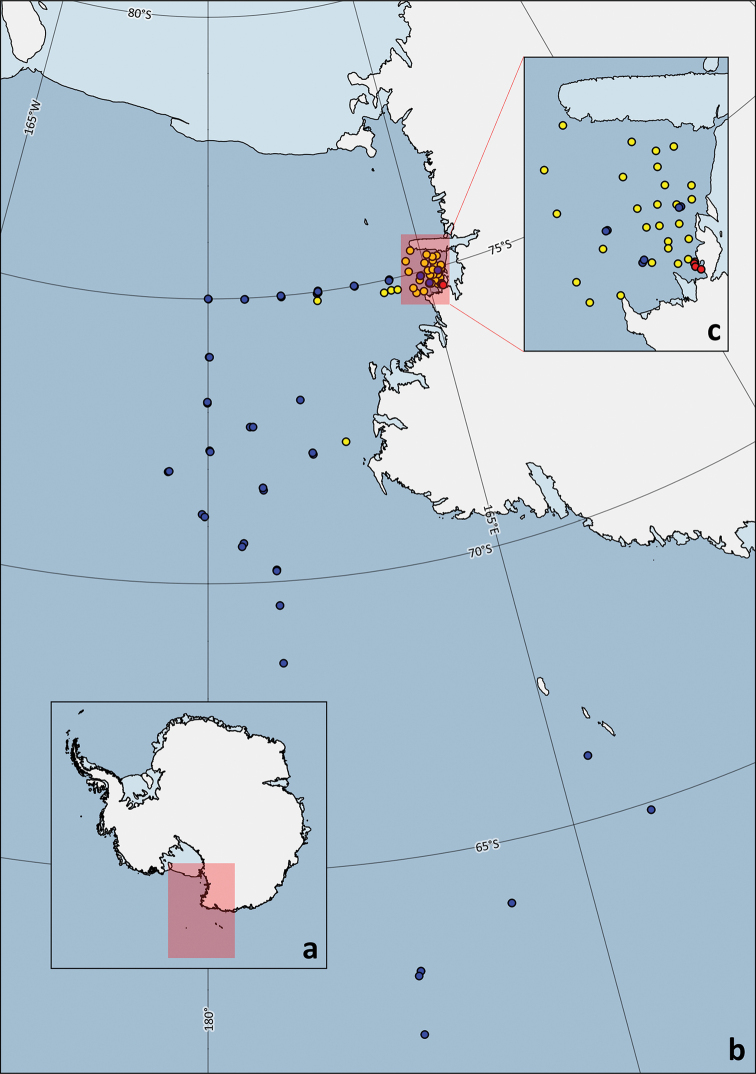
Sampling stations for III^rd^ (yellow), V^th^ (blue), and X^th^ (red) expedition **a** overview of spatial extent in Antarctica **b** sampling stations in the Western Ross Sea **c** focus on Terra Nova Bay sampling stations. This map was produced using the collection of datasets “Quantarctica” (Matsuoka et al. 2017) and QGIS (QGIS Development Team 2020).

As the distributional information provided in this data paper (Fig. 1) originates from three different PNRA expeditions that had a variety of scientific targets and research teams involved, the sampling stations included are only those where quantitative sampling methods were used and copepods were found.

For the III^rd^PNRA Expedition (first Italian Antarctic Expedition), 32 sampling stations located mainly in Terra Nova Bay between 72°S and 75°S of latitude and 163°E and 173°E of longitude (from 05/01/1988 to 21/02/1988) were investigated (Guglielmo et al. 1990). The V^th^PNRA Expedition (second Italian Antarctic Expedition) investigated a larger geographic area in the Pacific sector of the Southern Ocean, starting from 50°S to the Balleny Islands and finally reaching Terra Nova bay. The data included in this dataset correspond to 23 sampling stations surveyed between 62°S and 75°S and 161°E and 177°W (from 25/11/1989 to 12/1/1990) (Guglielmo et al. 1992). During this expedition, each station was sampled twice, and the duplicates were indicated as “bis” (e.g., Station 18/18bis). For the X^th^PNRA Expedition (third Italian Antarctic Expedition), five stations surveyed in the area surrounding “Mario Zucchelli” station (from 02/11/94 to 03/01/95) were included.

**Figure 2.**
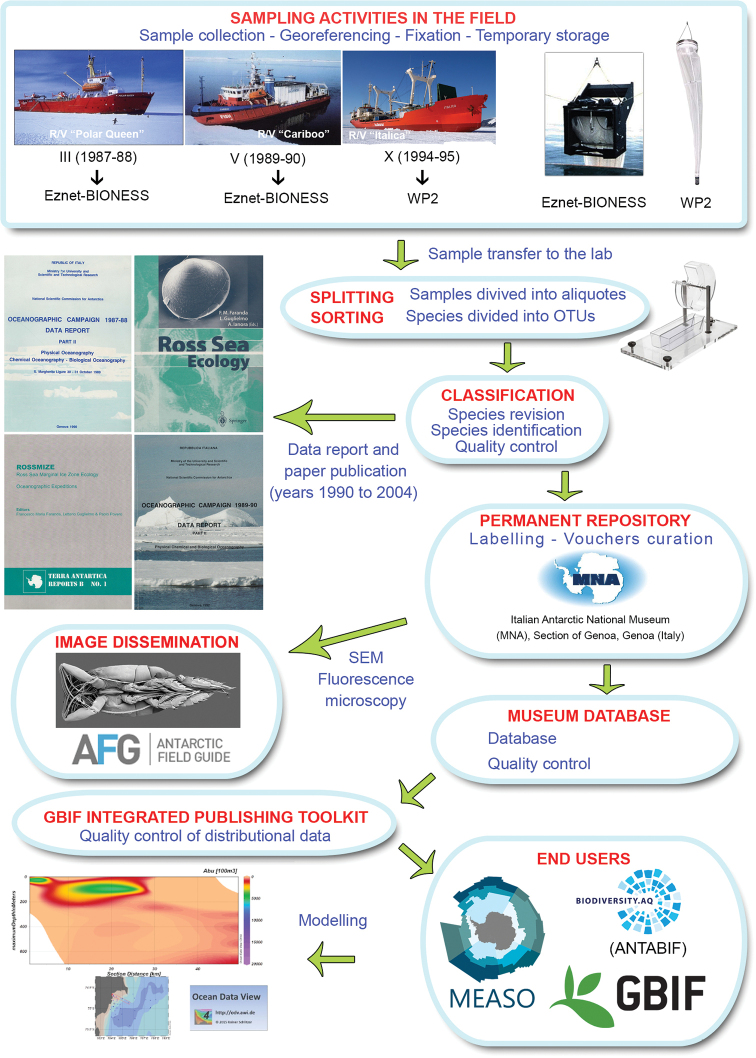
Flowchart representing all stages in dataset development and publishing.

The majority of mesozooplankton samples from this dataset (i.e., those from the III^rd^ and the V^th^PNRA Expeditions) were collected using an Eznet-BIONESS multi-net (Fig. 2) (Sameoto et al. 1980), a very efficient zooplankton sampler consisting of multiple (usually ten) nets, stacked horizontally, opened and closed at desired depths by an on-board operator while the instrument is towed from a vessel (ICES 2000). Due to the exceptional filtration to mouth area ratio (10:1), a 90% filtration efficiency can be reached for a clean net towed at 1.5 m/s.

The Eznet-BIONESS was equipped with a KMS II (ME Meerestechnik Elektronik GmbH) multiparametric probe (that recorded temperature, salinity, depth, light attenuation, and oxygen concentration) and two acoustic doppler flowmeters (SM 21H-ME Meerestechnik Elektronik GmbH), put inside and outside the filtering apparatus, that recorded speed, in- and out-flow through the net, filtration efficiency, and net number. Different mesh sizes were used during the sampling activity but, regarding Copepoda, only 500 µm and 250 µm sizes were considered.

Another sampling device employed during these PNRA activities (X^th^PNRA Expedition) was a Working Party II (WP2 – UNEP FAO) standardized net (Fraser 1966; ICES 2000). This net had a 57 cm (0.25 m^2^) opening, a length of 2.6 m, and a 200 µm mesh size. The WP2 was equipped with inner and outer General Oceanic flowmeter to the evaluation of the filtration efficacy. Sampling depths ranged from 200 m to the surface, depending on the sea-bottom depth of the sampling points (Carli et al. 2002).

More details about the sampling methodologies and procedures adopted during the III^rd^ and V^th^PNRA Expeditions can be found in Guglielmo et al. (1990, 1992).

All samples collected during the three campaigns were preserved on board in a 4% buffered formaldehyde seawater solution and later dispatched to various experts (see below) for determination. Specimens now present in the collections of the Italian National Antarctic Museum are stored in 96% Ethanol.

### Spatial coverage

**General geographic description**: The study area covers a large portion of the north-western Ross Sea, spanning from the Drygalski Ice Tongue in Terra Nova Bay to the continental slope surrounding the Central Basin. Some sampling stations were located at the Balleny Islands and other northern areas (Fig. 1).

**Coordinates**: Latitude bounding coordinates: -61.99067 and -75.40556; Longitude bounding coordinates: 161.82867 and -177.74167

### Temporal coverage

05 January 1988 to 11 February 1995.

### Dataset description and quality control

**Title**: Distributional records of Ross Sea (Antarctica) planktic Copepoda from bibliographic data and samples curated at the Italian National Antarctic Museum (MNA).

**Character encoding**: UTF-8;

**Format name**: Darwin Core Archive format;

**Distribution**: https://doi.org/10.15468/zndaaw

**Language**: English;

**Metadata language**: English;

**License of use**: This dataset [Distributional records of Ross Sea (Antarctica) planktic Copepoda from bibliographic data and samples curated at the Italian National Antarctic Museum (MNA)] is made available under the Creative Commons Attribution License (CC-BY) 4.0: http://www.creativecommons.org/licenses/by/4.0/legalcode

**Date of metadata creation**: 10 Feb. 2020;

This dataset comprises a total of 6,027 distributional records, out of which 5,306 were obtained by digitizing original data reports (hereafter “literature records”) and 721 are based on physical museum vouchers (hereafter “MNA collection records”) curated by the MNA (Section of Genoa).

All literature records (defined by the term ‘HumanObservation’ under the column ‘BasisOfRecord’) were manually extracted from five different data reports published in 1990, 1992, and 2002 (Carli et al. 1990, 1992, 2002; Zunini Sertorio et al. 1990, 1992). The information regarding the sampling events for the III^rd^ and V Italian Antarctic expeditions (e.g. sampling station coordinates, depth, volume filtered, etc.) was manually extracted from two other data reports (Guglielmo et al. 1990, 1992). The general characteristics of the sampling events are reported in the “Event” dataset with starting and ending coordinates (‘footprintWKT’ term) along with information on the subsequent samples handling (e.g., the examined aliquot) listed under the Darwin Core term ‘dynamicProperties’. All the MNA collection records (defined by the term ‘PreservedSpecimen’ under the column ‘BasisOfRecord’) correspond to a section of the entire batch of samples collected during the III^rd^ and V^th^ expeditions that were not previously sorted at the species level.

As the two different types of distributional data, i.e., MNA collection records and literature records, originated from the same sampling events, an apparent conflict might derive from multiple records sharing the same taxonomy, sampling event (i.e., sampling station, depth) and organism quantity information in the dataset. However, all the MNA collection records originated from plankton aliquots not previously studied and published, thus representing additional data that were not included by the original authors. Most of the literature records are reported with their original abundance values (number of individuals per volume unit, e.g. m^3^), whereas all the MNA collection records are reported in terms of number of individuals per museum vial. Some literature records from the V^th^ expedition were originally reported with the number of individuals (Zunini Sertorio et al. 1992), instead of an abundance measure. However, the number of individuals reported for these bibliographic records can indeed be converted in abundance measures, as the authors of the original bibliographic reference provided the volumetric information on the aliquot examined.

All data were then gathered in a single dataset formatted to fulfil the Darwin Core standard protocol (Wieczorek et al. 2012) required by the OBIS scheme (http://www.iobis.org/manual/lifewatchqc/) and according to the SCAR-MarBIN Data Toolkit (http://www.scarmarbin.be/documents/SM-FATv1.zip). The dataset was uploaded and integrated with the ANTOBIS database (the geospatial component of SCAR-MarBIN). The taxonomy was checked and updated using WoRMS (Horton et al. 2019, World Register of Marine Species; http://www.marinespecies.org; last accessed 02 December 2019). Different control and data-cleaning steps (e.g., scientific name check and spelling) were undertaken to increase data quality (Fig. 2).

The Darwin Core elements included in the dataset are: occurrenceID, BasisOfRecord (HumanObservation for the bibliographic records and PreservedSpecimen for the museum specimen records), type (identifying the nature of the resource), scientificName (the name in the lowest taxonomic rank identified and updated according to WoRMS with authorship and date for the records identified at the species level), order, family, genus, specificEpithet, scientificNameAuthorship (corresponding to the updated taxonomy according to WoRMS, together with the previous four elements), originalNameUsage (the original identification as reported in the bibliographic resource), identificationQualifier (the qualifier for the uncertainty of identification, following Sigovini et al. 2016), scientificNameID (the globally unique identifier for the taxonomic information related to the scientificName and stored in WoRMS), taxonRemarks (notes and considerations regarding the taxonomy of the record), organismQuantity, organismQuantityType (the type of quantification system used, such as the number of individuals or abundance per 100 or one cubic metre), sex, lifeStage (following the controlled vocabulary ‘BODC parameter semantic model biological entity development stage terms’ at https://github.com/nvs-vocabs/S11), occurrenceRemarks (name of the PNRA research expedition), fieldNumber (name of the sampling station and net number, separated by an underscore), eventDate (date of the sampling event), decimalLatitude, decimalLongitude, minimumDepthInMeters, maximumDepthInMeters, sampleSizeValue (the number of cubic meters filtered by the net as reported in the bibliographic resource), sampleSizeUnit, samplingProtocol (following the controlled vocabulary at http://vocab.nerc.ac.uk/collection/B07/current/, Wiebe et al. 2014), eventRemarks (name of the sampling gear as reported in the bibliographic reference and mesh size of the net, separated by a pipe), associatedReferences (bibliographic reference associated to the resource), preparations (following ‘Documentation for code table SPECIMEN_PART_NAME’ at http://arctos.database.museum/info/ctDocumentation.cfm?table=CTSPECIMEN_PART_NAME), catalogNumber (museum voucher code for the specimen). Most of the sampling stations have two sets of coordinates: the starting and ending points. In such cases, the coordinates reported in the dataset refer to the starting point of the sampling event. For some museum records, the net number, which corresponds to a specific depth stratum investigated, was not available. For these records, the minimum depth was omitted, while for the maximum depth the value recorded for the corresponding sampling station was reported.

### Taxonomic coverage

**Figure 3.**
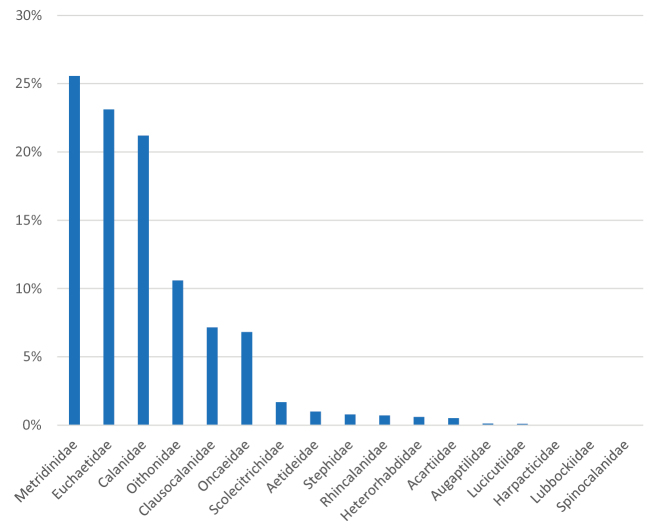
Taxonomic diversity for data report analysis.

**Figure 4.**
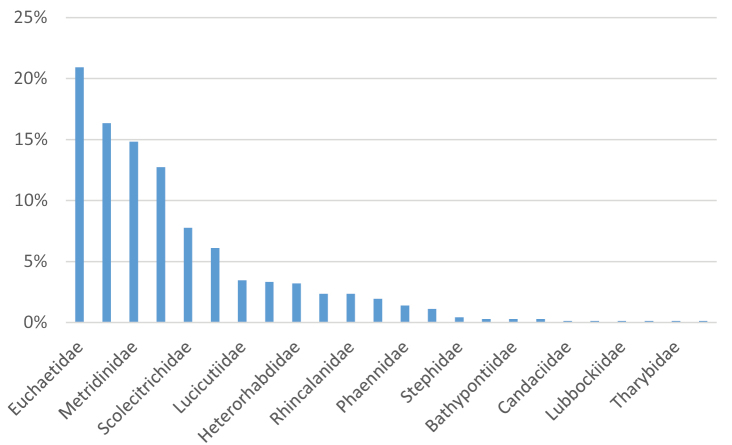
Taxonomic diversity for museum vouchers.

The Copepoda diversity of the dataset is displayed in 6,027 records, among which Calanoida represent the most frequent (80%), followed by Cyclopoida (15.3%) and unidentified Copepoda (4.6%). Only five records belong to Harpacticoida and one to Siphonostomatoida. Regarding the life stages identification, the data set is composed of most adults (52.4%), followed by copepodites (45.5%), nauplii (0.7%), and unreported (1.4%). The three campaigns (III^rd^, V^th^, and X^th^) data report analysis produced a combined total of 5,306 literature reports divided among 52 morphological units. Among these, 26 species belong to three orders (Calanoida, Cyclopoida, Harpacticoida) and 17 families. Overall, Calanoida were the most frequently found (78.5%), followed by Cyclopoida (16.25%) and Harpacticoida (0.07%). In terms of sampling frequency, among the determined specimens, members of family Metridinidae were the most common (25.56%), followed by Euchaetidae (23.11%), Calanidae (21.19%), and Oithonidae (10.6%); the other 14 families accounted for the remaining 19.53% (Fig. 3). Overall, 721 museum vouchers were acquired from the National Antarctic Museum (MNA collection records), among which Calanoida represented the major contributors (91.6%), followed by Cyclopoida (8.2%) and two records belonging to Harpacticoida and Siphonostomatoida, respectively. Calanoid diversity spans 19 families, among which we find a relevant percentage of Euchaetidae (22.8%), Calanidae (17.8%), Metridinidae (16.2%), Aetideidae (13.9%), Scolecithridae (8.5%), Lucicutiidae (3.8%), and others (17%). Cyclopoida diversity accounts for three families and four genera, while Harpacticoida and Siphonostomatoida are represented by one species each (Fig. 4).

## Taxonomic rank

**Kingdom**: Animalia

**Phylum**: Arthropoda

**Class**: Maxillopoda

**Order**: Calanoida, Cyclopoida, Harpacticoida, Siphonostomatoida

**Families**: Acartiidae, Aetideidae, Augaptilidae, Bathypontiidae, Calanidae, Candaciidae, Clausocalanidae, Eucalanidae, Euchaetidae, Harpacticidae, Heterorhabdidae, Lubbockiidae, Lucicutiidae, Metridinidae, Oithonidae, Oncaeidae, Paracalanidae, Phaennidae, Rataniidae, Rhincalanidae, Scolecitrichidae, Spinocalanidae, Stephidae, Tharybidae, Tisbidae

**Genera**: *Aetideopsis*, *Aetideus*, *Amallothrix*, *Calanoides*, *Calanus*, *Calocalanus*, *Candacia*, *Cephalophanes*, *Chiridiella*, *Chiridius*, *Chirundina*, *Clausocalanus*, *Cornucalanus*, *Ctenocalanus*, *Euaugaptilus*, *Eucalanus*, *Euchirella*, *Farrania*, *Gaetanus*, *Haloptilus*, *Harpacticus*, *Heterorhabdus*, *Lubbockia*, *Lucicutia*, *Metridia*, *Microcalanus*, *Oithona*, *Oncaea*, *Onchocalanus*, *Paracalanus*, *Paracomantenna*, *Paraeuchaeta*, *Paraheterorhabdus*, *Paralabidocera*, *Phaenna*, *Pleuromamma*, *Pontoptilus*, *Pseudeuchaeta*, *Pseudhaloptilus*, *Pseudoamallothrix*, *Pseudochirella*, *Racovitzanus*, *Ratania*, *Rhincalanus*, *Scaphocalanus*, *Scolecithricella*, *Spinocalanus*, *Stephos*, *Temorites*, *Tisbe*, *Triconia*, *Undinella*

**Species**: *Aetideopsis antarctica*, *Aetideopsis minor*, *Aetideus australis*, *Aetideus pseudarmatus*, *Amallothrix gracilis*, *Amallothrix dentipes*, *Calanoides acutus*, *Calanoides carinatus*, *Calanus propinquus*, *Candacia falcifera*, *Cornucalanus robustus*, *Ctenocalanus vanus*, *Euaugaptilus laticeps*, *Euchirella rostromagna*, *Euchirella rostrata*, *Farrania frigida*, *Gaetanus tenuispinus*, *Gaetanus inermis*, *Gaetanus brevispinus*, *Gaetanus minor*, *Haloptilus ocellatus*, *Harpacticus furcifer*, *Heterorhabdus austrinus*, *Heterorhabdus pustulifer*, *Heterorhabdus tanneri*, *Lucicutia ovalis*, *Lucicutia wolfendeni*, *Lucicutia magna*, *Lucicutia intermedia*, *Lucicutia curta*, *Lucicutia macrocera*, *Metridia gerlachei*, *Metridia curticauda*, *Microcalanus pygmaeus*, *Oithona frigida*, *Oithona similis*, *Oncaea curvata*, *Onchocalanus magnus*, *Paraeuchaeta antarctica*, *Paraeuchaeta similis*, *Paraeuchaeta exigua*, *Paraeuchaeta comosa*, *Paraeuchaeta kurilensis*, *Paraheterorhabdus farrani*, *Paralabidocera antarctica*, *Pleuromamma robusta*, *Pleuromamma gracilis*, *Pleuromamma abdominalis*, *Pontoptilus ovalis*, *Pseudhaloptilus eurygnathus*, *Pseudoamallothrix ovata*, *Pseudochirella hirsuta*, *Pseudochirella notacantha*, *Racovitzanus antarcticus*, *Ratania atlantica*, *Rhincalanus gigas*, *Scaphocalanus subbrevicornis*, *Scaphocalanus magnus*, *Scaphocalanus vervoorti*, *Scaphocalanus affinis*, *Scaphocalanus brevicornis*, *Scolecithricella minor*, *Spinocalanus abyssalis*, *Spinocalanus magnus*, *Spinocalanus horridus*, *Spinocalanus brevicaudatus*, *Stephos longipes*, *Temorites brevis*, *Triconia conifera*, *Triconia antarctica*, *Undinella simplex*

### History of the Copepoda collection

The Antarctic copepods sampled during the three expeditions were studied by different research groups and experts in different times. The III^rd^ expedition samples were determined and studied by T.Z. Sertorio, P. Salemi Picone, P. Bernat, E. Cattini, C. Ossola, A. M. Carli, L. Pane, and G.L. Mariottini (Carli et al. 1990; Zunini Sertorio et al. 1990). Samples from the V^th^ expedition were examined by T.Z. Sertorio, P. Licandro, F. Ricci, M. Giallain, A. Artegiani, L. Pane, A. Carli, G.L. Mariottini, and M. Feletti (Carli et al. 1992; Zunini Sertorio et al. 1992). Samples from the X^th^ expedition were studied by L. Pane, A. Carli, M. Feletti , and B. Francomacaro (Pane et al. 2004).

Other samples from the V^th^ expedition were also studied and later published by (Zunini Sertorio et al. 2000). In this case, with the aid of Dr Elena Markhaseva and Dr Nina Vyskvartzeva of the Zoological Institute of the Russian Academy of Sciences of St Petersburg, new determinations and records of species were added. However, these records were grouped by family and reported only with a general indication of the sampling station without details about depth or abundance. The fate of these samples is unknown and are therefore not available in the MNA collections. For this reason, these latter records were not included in the present dataset but just listed here with the classification reported in the original paper (Zunini Sertorio et al. 2000):

*Mimocalanus cultrifer* (Farran, 1908); *Mimocalanus inflatus* (Davis, 1949); *Spinocalanus antarcticus* (Wolfenden, 1906); *Spinocalanus spinipes* (Brodsky, 1950); *Spinocalanus spinosus* (Farran, 1908); *Chiridiella megadactyla* (Bradford, 1971); *Gaetanus antarcticus* (Wolfenden, 1905); *Pseudochirella elongata* (Wolfenden, 1905); *Cornucalanus antarcticus* (Brodsky & Zvereva, 1950); *Lophotrix simplex* (Wolfenden, 1911); *Mixtocalanus alter* (Farran, 1929); *Mixtocalanus vervoorti* (Park, 1980); *Scaphocalanus antarcticus* (Park, 1982); *Scaphocalanus echinatus* (Farran, 1905); *Scaphocalanus farrani* (Park, 1982); *Scaphocalanus parantarcticus* (Park, 1982); *Scolecithricella cenotelis* (Park, 1980); *Scolecithricella dentipes* (Vervoort, 1951); *Scolecithricella emarginata* (Farran, 1905); *Scolecithricella ovata* (Farran, 1905); *Temora* sp.; *Undinella acuta* (Vaupel-Klein, 1970); *Hemirhabdus* sp.; *Heterostylites longicornis* (Giesbrecht, 1889); *Euaugaptilus antarcticus* (Wolfenden, 1911); *Euaugaptilus nodifrons* (Sars, 1905); *Haloptilus oxycephalus* (Giesbrecht, 1889); *Pachyptilus pacificus* (Johnson, 1936).

Finally, the whole MNA copepod collection was recently reorganized and taxonomically revised at the lowest possible taxonomic level for the present contribution by G. Bonello and M. Grillo under a research contract with the MNA. For this final check, species identifications were based on the Banyuls sur Mer marine Copepoda database (Razouls et al. 2020; https://copepodes.obs-banyuls.fr), while the current taxonomical state was cross-checked with the Register of Antarctic Marine Species, RAMS (De Broyer et al. 2020) (last accessed 02 December 2019) . For all 82 species present in the MNA collections, a collection of permanent slides for microscopy were prepared by mounting specimens within plastic adhesive rings, filled with Glycerol, to avoid undesired flattening. When possible, multiple specimens were acquired in both ventral and lateral view to ease eventual future analysis (Kihara and da Rocha 2009). Transparent varnish was used to seal the slides after mounting them.

### Copepod image acquisition

For all the species listed in this data paper, a selection of complete specimens was prepared to produce high-quality images and highlight taxonomical characters necessary for species identification. For this purpose, different imaging techniques were applied: i) Scanning Electron Microscopy (SEM) after gold coating (e.g., for *Paraeuchaeta exigua* (Wolfenden, 1911), Fig. 5); ii) fluorescence microscopy after staining. In this latter case, Copepoda specimens were stained with 1.5 mg/ml Congo Red solution following the methods of Michels and Büntzow (2010) and with different Congo Red and Fuchsin dilutions (Ivanenko et al. 2012). Images were then acquired with an Olympus IX70 (200×) inverted microscope (e.g., *Metridia gerlachei* (Giesbrecht, 1902), Fig. 6) provided with a fluorescent light apparatus. Image post-processing and cleaning were performed with Adobe Photoshop CC 2015 and Nik Collection filters (Sharpener and Silver Efex).

**Figure 5.**
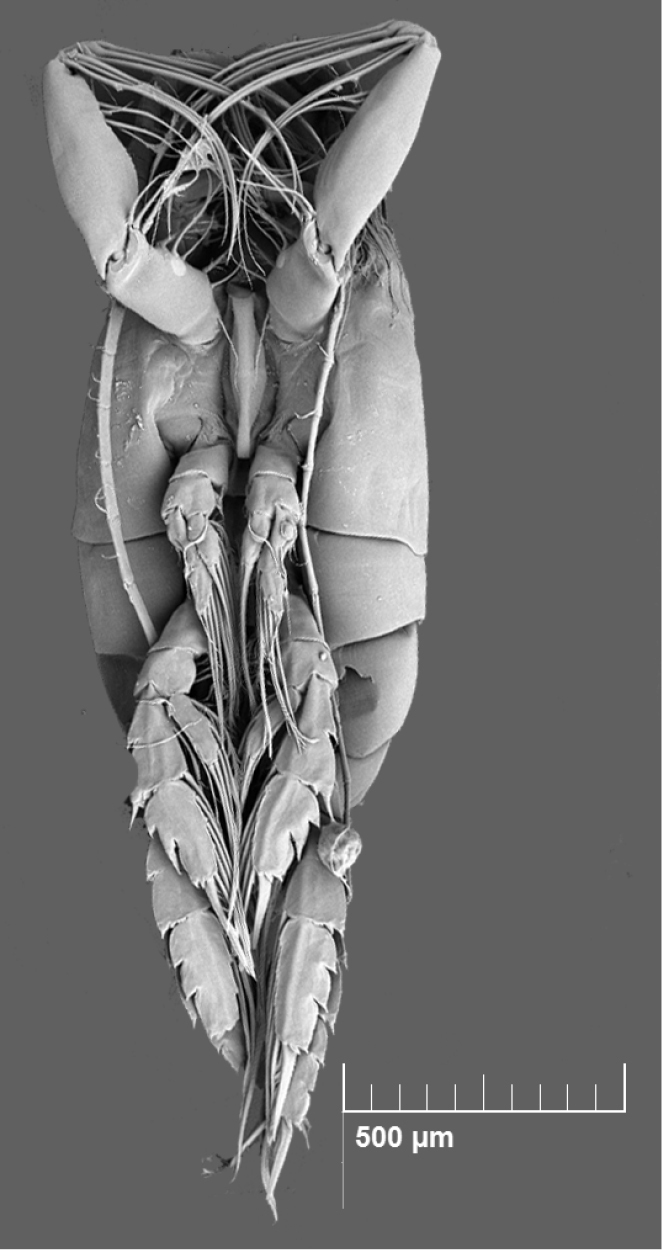
*Paraeuchaeta exigua* (Copepoda, Calanoida; female, MNA-12333) acquired with scanning electron microscopy (SEM). This is one of the most common species in the coastal area of Terra Nova Bay. It plays a key role in the neritic trophic chain and highly contributes to the total mesozooplanktic biomass.

### Geographic data and new distributional records

To evaluate the number of potential new records for a given area, defined as new occurrences in the Global Biodiversity Information Facility repository (GBIF, https://www.gbif.org) for that area, we have used the *spocc* (version 1.0.8) R package, as well as the online Copepod database provided by the Banyuls sur mer observatory (Razouls et al. 2020; https://copepodes.obs-banyuls.fr). For the analysis with *spocc* we selected the sources ‘gbif’, ‘obis’, ‘ecoengine’, ‘inat’, ‘idigbio’ and produced a distributional map for each single species. The Banyuls sur mer observatory interactive database gathers information on diversity and distribution of planktic Copepoda from the available literature and is continuously updated with the latest research.

**Figure 6.**
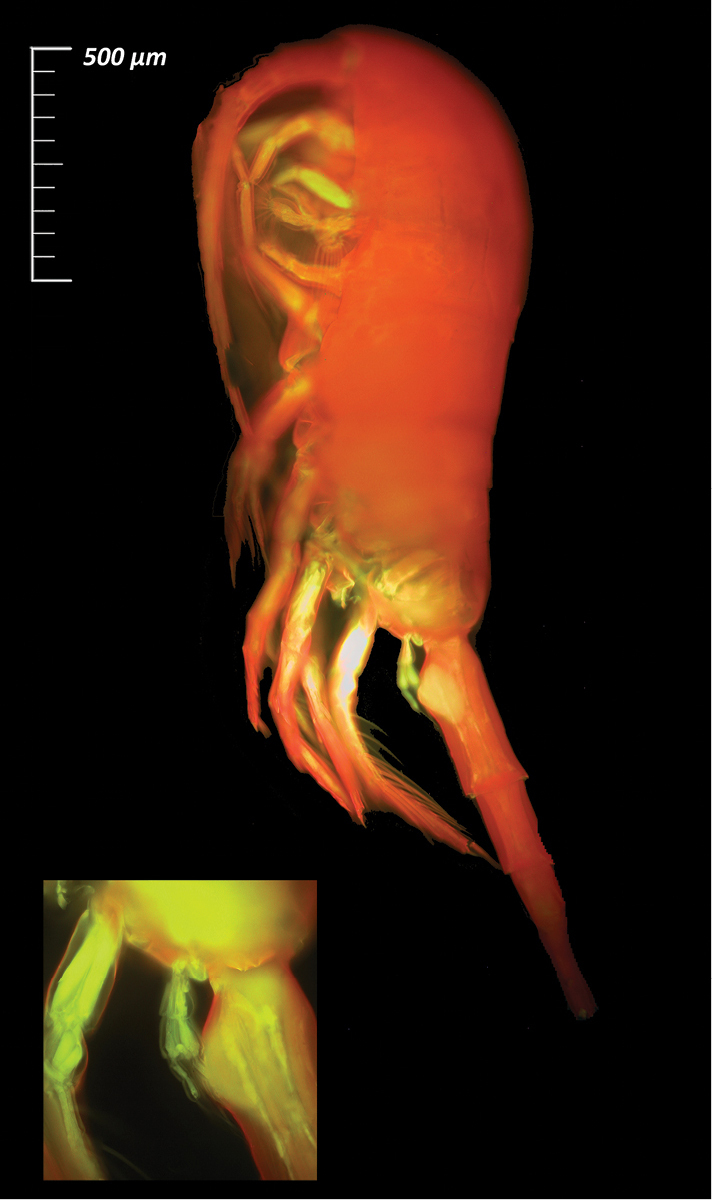
*Metridia gerlachei* (Copepoda, Calanoida; female, MNA-12439) acquired with fluorescence microscopy (Congo Red, 1.5 mg/ml). This species is one of the most adapted species in the Antarctic region and can perform diel vertical migrations that highly influence the surrounding waters in terms of trophic relationships in Terra Nova Bay.

To our knowledge, regarding the Ross Sea area and its boundaries, 62% of the species reported (*n* = 71) in this data paper represent new records for GBIF for the Western Ross Sea sector and 28% for the whole Antarctic region. It must be considered that some of the sampling stations were close to the northern boundaries of the circumpolar current and the Ross Sea Gyre, hence the presence of pelagic copepods typical for sub-Antarctic areas.
